# A randomized trial of artemether-lumefantrine *versus *mefloquine-artesunate for the treatment of uncomplicated multi-drug resistant *Plasmodium falciparum *on the western border of Thailand

**DOI:** 10.1186/1475-2875-4-46

**Published:** 2005-09-22

**Authors:** Robert Hutagalung, Lucy Paiphun, Elizabeth A Ashley, Rose McGready, Alan Brockman, Kaw L Thwai, Pratap Singhasivanon, Thomas Jelinek, Nicholas J White, François H Nosten

**Affiliations:** 1 Shoklo Malaria Research Unit, Mae Sod, Tak Province, Thailand; 2 Faculty of Tropical Medicine, Mahidol University, Bangkok, Thailand; 3 Centre for Vaccinology and Tropical Medicine, Nuffield Department of Clinical Medicine, John Radcliffe Hospital, Headington, Oxford, UK; 4 Menzies School of Health Research, Darwin, NT, Australia; 5 Institut für Tropenmedizin, Berlin, Germany

## Abstract

**Background:**

The use of antimalarial drug combinations with artemisinin derivatives is recommended to overcome drug resistance in *Plasmodium falciparum*. The fixed combination of oral artemether-lumefantrine, an artemisinin combination therapy (ACT) is highly effective and well tolerated. It is the only registered fixed combination containing an artemisinin. The trial presented here was conducted to monitor the efficacy of the six-dose regimen of artemether-lumefantrine (ALN) in an area of multi-drug resistance, along the Thai-Myanmar border.

**Methods:**

The trial was an open-label, two-arm, randomized study comparing artemether-lumefantrine and mefloquine-artesunate for the treatment of uncomplicated falciparum malaria with 42 days of follow up. Parasite genotyping by polymerase chain reaction (PCR) was used to distinguish recrudescent from newly acquired *P. falciparum *infections. The PCR adjusted cure rates were evaluated by survival analysis.

**Results:**

In 2001–2002 a total of 490 patients with slide confirmed uncomplicated *P. falciparum *malaria were randomly assigned to receive artemether-lumefantrine (n = 245) or artesunate and mefloquine (n = 245) and were followed for 42 days. All patients had rapid initial clinical and parasitological responses. In both groups, the PCR adjusted cure rates by day 42 were high: 98.8% (95% CI 96.4, 99.6%) for artemether-lumefantrine and 96.3% (95% CI 93.1, 98.0%) for artesunate-mefloquine. Both regimens were very well tolerated with no serious adverse events observed attributable to either combination.

**Conclusion:**

Overall, this study confirms that these two artemisinin-based combinations remain highly effective and result in equivalent therapeutic responses in the treatment of highly drug-resistant falciparum malaria.

## Introduction

Multi-drug resistance of *Plasmodium falciparum *is a major health problem in many countries and the number of drugs available, effective and affordable is very limited [1]. Along the Thai-Myanmar border, *P. falciparum *has developed resistance to almost all available antimalarials [2]. As in tuberculosis and HIV where resistance to drugs is a serious issue, combination therapy has been applied to malaria treatment [3]. The use of antimalarial drug combinations with artemisinin derivatives has been advocated and is now implemented in many countries [4]. An extensive amount of information on efficacy and safety of mefloquine has been reported and reviewed [5]. Artemisinin or Qinghaosu is an extract of the medical plant Qinghao (*Artemisia annua*), which together with its derivatives, artesunate and artemether are the most active antimalarial compounds to date [6]. The artemisinin derivatives have a rapid onset of therapeutic effect, where a single dose can reduce the parasite biomass by a factor of approximately 10^4 ^every 48 hours. In addition, they have a very short terminal elimination half-life of less than 2 hours [7]. Previous studies showed that once-daily administration with artemisinin derivatives produced equivalent cure rates to more frequent administration [8]. A three-day course of artesunate combined with high dose mefloquine has become the standard treatment combination for *P. falciparum *infections in Thailand [9]. Oral artesunate-mefloquine is the most widely used combination. More recently, a fixed combination of oral artemether-lumefantrine (formerly known as benflumetol) has become available. Artemether is a methyl-ether derivative of artemisinin. Lumefantrine is a racemic fluorine derivative with high blood schizontocidal activity [10]. Both artemisinin combination therapies (ACT) are highly effective and well tolerated [11]. However, resistance to mefloquine and/or to lumefantrine, would compromise both combinations. Therefore it is important to monitor the therapeutic efficacy and thus provide advance warning in case of change. The trial presented here was conducted to monitor the efficacy of the six-dose regimen of artemether-lumefantrine combination given over three days for the treatment of uncomplicated *P. falciparum *infections in adults and children on the western border of Thailand.

## Patients and methods

This study was conducted in the Maela and Mawker Tai malaria clinics of the Shoklo Malaria Research Unit (Mae Sot, Thailand)between July 2001 and June 2002. Patients were recruited from two populations: displaced people of the Karen ethnic minority and migrant workers living on the western border of Thailand. This is an area of low and unstable transmission of *Plasmodium vivax *and multi-drug-resistant *P. falciparum *[12]. The trial was an open-label, two-arm, randomized study comparing artemether-lumefantrine andmefloquine-artesunate. This study was approved by the Ethical and Scientific Committees of the Faculty of Tropical Medicine, Mahidol University.

### Procedures

Patients >10 kg in weight who had slide-confirmed acute *P. falciparum *malaria were included in the study, provided that they or their guardians gave fully informed written consent intheir own language, they were not pregnant, they had not received mefloquine in the previous 63 days and there were no other clinical or laboratory signs of severe illness and/or severe and complicated malaria [13]. If they gave written informed consent, they were allocated randomly to receive either the six-dose regimen of artemether-lumefantrine (Coartem^® ^20/120, Novartis Pharma AG, Basel, Switzerland) or mefloquine (Lariam^®^, Hoffman-La Roche, Basel, Switzerland) plus artesunate (Guilin Pharmaceutical Factory No.1, Guilin, China). At enrolment (Day 0), a medical history was obtained, a full physical examination was performed and blood was taken for quantitative parasite counts and routine haematology (finger prick blood sample for malaria smear and haematocrit). All information was recorded on a standard case record form. All patients were examined and blood smears were taken daily until they became aparasitaemic, and then weekly for 6 weeks. At each visit a questionnaire on adverse events was completed. A blood smear was also taken from any patient complaining of fever or symptoms compatible with malaria during the follow-up period. Parasite counts were determined on Giemsa-stained thick and thin blood films. The person-gametocyte-weeks were calculated per 1,000 person-weeks after excluding the episodes on admission and during treatment.

### Drug regimens

Computerized randomization was in blocks of ten. Patients allocated to artemether-lumefantrine group (ALN) received the tablets at 0 and 8 hours and twice daily for the following 2 days. Artemether-lumefantrine was dispensed as a fixed dose combination tablet. Each tablet contained 20 mg of artemether and 120 mg of lumefantrine. The number of tablets was given according to the body weight. The minimum dosage for patients weighing less than 15 kg was one tablet per dose; patients between 15 and 24 kg received two tablets, those between 25 and 34 kg received three tablets and patients 35 kg and above were treated with four tablets per dose. Patients allocated to artesunate-mefloquine group (MAS3) received artesunate, 4 mg/kg oncedaily for 3 days (day 0 was the first day of treatment), plus mefloquine, 15 mg/kg on day 1 and 10 mg/kg on day 2.

Each patient was given antipyretics and cooled by tepid sponging if the tympanic temperature was equal or above 37.5°C before drug administration. Drug administration was observed in all patients and if vomiting occurred in less than 30 min, administration of the full dose was repeated, if vomiting occurred between 30 and 60 min, half the dose was repeated. Patients treated with artemether-lumefantrine were given a glass of chocolate milk (200 ml) with each dose to increase absorption [14].

### Outcome measures

The primary therapeutic outcome measure in this study was the incidence of microscopically and genotypically confirmedrecrudescent infections in both treatment groups by day 42. Parasite genotyping by the polymerase chain reaction (PCR) was used to distinguish recrudescent from newly acquired *P. falciparum *infections. *P. falciparum *infections were genotyped for allelic variation in three polymorphic antigen loci, merozoite surface proteins 1 and 2 (MSP-1 and MSP-2) and glutamate rich protein (GLURP), on admission and in case of parasite reappearance [15,16]. Secondary measures were the immediate treatment responses: parasite clearance, fever clearance, incidence of adverse events, and degree of anaemia. The sample size was calculated to detect a difference in failure rates of 7 % between the two regimens with 90% CI and 80 % power assuming a 20% drop out.

### Adverse events

Adverse events were symptoms or signs that were not presenton admission and that developed after the start of treatment. All adverse events, including those probably related to malaria, were recording and compared among treatment groups. The rates of early vomiting (<1 h) after each dose and for each drug wererecorded and compared among the groups in the analysis.

### Management of recrudescent infections

Patients with uncomplicated recrudescent infections were re-treated with artesunate, 2 mg/kg/day for 7 days; patients >8 years oldalso received doxycycline, 4 mg/kg/day for 7 days.

### Statistical analysis

Data were analysed using SPSS for Windows, version 11. Categorical data were compared using the Chi-square test with Yates' correction or by Fisher's exact test, as appropriate. Continuous variables conforming to a normal distribution were compared using Student's *t *test. Data not normally distributed were log-transformed or compared using the Mann-Whitney *U *test. The relative risks were calculated using cross-tabulations. The rates of adverse events at three different periods (days 1–2, days 3–7 and days 14–42) were compared among treatment groups. For each of the three periods, the events were counted only once (e.g., if a patient vomited on day one and day two, this was counted as one adverse event). The PCR-adjusted cure rates were evaluated by survival analysis and compared using the log-rank test. Patients for whom PCR genotyping was either inconclusive or missing were censored in the survival analysis on the day of parasite reappearance. For all statistical tests the significance level (p) was set at 0.05.

## Results

Four hundred and ninety patients with uncomplicated *P. falciparum *infections were enrolled between July 2001 and May 2002. The artesunate-mefloquine and artemether-lumefantrine groups included 245 patients each, the age range for all patients was 2–72 years. Baseline characteristics were similar in both groups (Table 1). In total, 484 patients (242 each group) were included in the final evaluation. Six patients (three in each group) were excluded for the following reasons; withdrew consent (1), non-compliance e.g. failure to complete trial treatment course (4), failure to meet protocol criteria (1). Overall compliance was good; around 99% of the patients in the study (481 of 484) were seen at the day seven scheduled visit, 96.1% (465 patients) were seen at day 28 and 93.4% (452 patients) were seen at day 42.

**Table 1:** Demographic and baseline characteristics

	ALN* (n = 245)	MAS3** (n = 245)
Maela	100	100
Mawker Tai	145	145
Males, no. (%)	172 (70)	164 (67)
Age, years		
Mean (SD)	23.2 (14.6)	23.6 (15.1)
Range	3–70	2–72
Age group, no. (%)		
<5	10 (4.1)	8 (3.3)
5–14	81 (33.1)	75 (30.6)
>14	154 (62.9)	162 (66.1)
Weight, kg		
Mean (SD)	42.4 (14.5)	42.3 (15.0)
Range	10–77	10–78
Temperature, °C		
Mean (SD)	37.7 (1.0)	37.8 (1.1)
Range	35.6–40.5	35.9–41.0
Fever^a^, no (%)	136 (55.5)	142 (58.0)
Haematocrit, %		
Mean (SD)	36.7 (5.9)	36.4 (5.8)
Range	21–52	20–51
Geometric mean (range)	8,047	7,570
parasite count (μl^-1^)	(32–198,789)	(16–198,789)
Hepatomegaly, no (%)	48 (19.6)	50 (20.4)
Splenomegaly, no (%)	70 (28.6)	57 (23.3)

* ALN = artemether-lumefantrine, ** MAS3 = artesunate-mefloquine ^a^Tympanic temperature ≥ 37.5°C

### Clinical and parasitological findings

**Figure 1:**
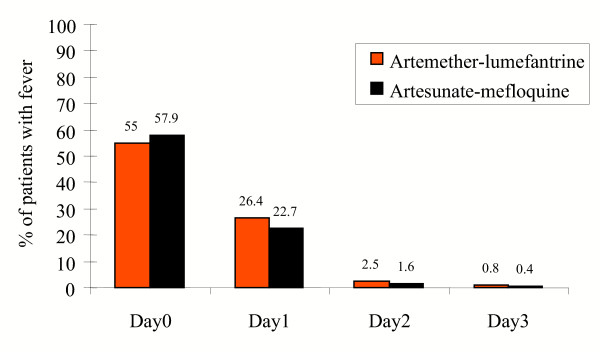
Percentage of patients with fever (temperature > 37.5°C).

**Figure 2:**
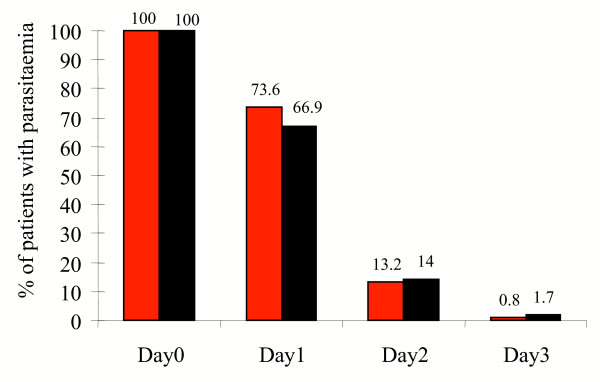
Percentage of patients with positive slide for asexual *P. falciparum *forms.

The initial responses to the two treatment groups were similar. None of the patients developed severe malaria. On admission, 55.0% (133/242) of the patients on ALN and 57.9% (140/242) of the patients in the MAS3 group had a tympanic temperature ≥ 37.5°C. All except three patients had a normal temperature on day 3 (2 in ALN and 1 in MAS3). There was no difference in fever clearance times between the two treatment groups (Figure 1). Parasite clearance times were short and most patients cleared their parasitaemia by day two. Figure 2 shows the percentage of patients with positive slide for asexual *P. falciparum *in both groups. By day three, four (1.8%) of 227 patients in the artemether-lumefantrine recipients and three (1.3%) of 238 artesunate-mefloquine recipients still had a positive blood film (*P *> 0.05). Overall, 12.3 % of patients were anaemic (haematocrit <30%) on admission, 10.8% in ALN group and 13.8% in MAS3 group (*P *= 0.33). The mean (SD) decrease in haematocrit value at day seven from baseline was greater in the group receiving MAS3 than in the ALN group: 9.3% (SD,11.5%; 95% CI, 7.7% to 10.9%) compared with 6.7% (SD, 11.4%; 95% CI, 5.1 to 8.3%) respectively (*P *= 0.023).

During the 42-day follow-up period, 27 new *P. falciparum *infections occurred among artemether-lumefantrine and 24 among artesunate-mefloquine recipients (*P *> 0.05) (Table 2). The PCR-adjusted cure rates by day 42 were 98.8% (95% CI, 96.4% to 99.6%) in the ALN group and 96.3% (95% CI, 93.1% to 98.0%) in the MAS3 group (*P *= 0.08). PCR confirmed treatment failures were more likely in children aged below 15 years than in adults (RR, 5.1; 95% CI, 1.4–18.7; *P *= 0.006). The mean age was 13.6 years (n = 12; SD = 8.5) in patients with treatment failure and 23.7 years (n = 438; SD = 15.2) in successfully treated patients. In this trial, only age group was independently associated with treatment failure, but not other factors e.g. higher parasitaemia (>10,000/μL); anaemia (haematocrit <30%); fever (tympanic temp ≥ 37.5°C) on admission; sites; treatment groups; early vomiting (within one hour following drug administration). The mediantime to recrudescence was comparable for MAS3 group (21 days; n = 9; range, 14–28 days) and ALN group (28 days; n = 3; range, 21–42 days; *P *> 0.05).

### Other parasitological findings

Of 452 patients, 119 (26.3%) had *P. vivax *parasitaemia detected during follow up. There were significantly fewer cases of vivax malaria in the MAS3 group (29 of 227) than in the ALN group (90 of 225) (*P *< 0.001). The median time to appearance of *P. vivax *parasitaemia was longer in the MAS3 group (40 days; range, 13–43 days) than in the ALN group (28 days; range, 14–43 days; *P *< 0.001).

Twenty patients (8.3%) in the artemether-lumefantrine group and 19 (7.9%) in the artesunate-mefloquine group had gametocytes detected during the first 3 days. All except one patient in ALN group cleared gametocytes within first week after start of treatment. After excluding these, gametocytes developed (between day 7 and 42) in 1.2% (3/241) of ALN group and 1.3 (3/240) of MAS3 group. The person-gametocyte weeks were low and similar: 2.7 (95% CI, 0.6–7.8) per 1000 person-weeks for both groups.

### Adverse events

Both treatment regimens were well tolerated. No serious adverse events were reported. Overall, 5/242(2.1%) of the patients vomited one or more doses of medication in the ALN group and 2/242(0.8%) of the MAS3 treated patients (RR, 2.5; 95% CI, 0.5–12.7; *P *= 0.45). The rates of early vomiting (within one hour) of the drugs were very low (around 2%) and did not differ among groups (one in each group on day 2).

**Figure 3:**
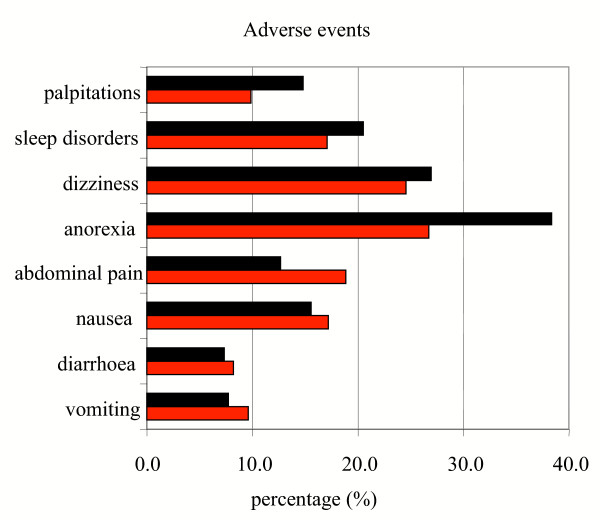
Possibly drug-related adverse events (day 1 – day 42).

The most commonly reported and possibly drug-related adverse events to both combination therapies were effects on the gastrointestinal (abdominal pain, anorexia, nausea, diarrhoea and late vomiting e.g. >1 h after administration of treatment) and central nervous system (headache, dizziness). Figure 3 shows the proportions of possibly drug-related adverse events of those who did not have those symptoms at admission during follow-up in both groups. Overall, there were less adverse events in ALN group compared to MAS3, though the differences were not statistically significant.

## Discussion

The loss of affordable effective antimalarial drugs to resistance represents a major threat to the people of malaria endemic countries [1]. Using ineffective drugs with high failure rates kills many and is unacceptable. A clear treatment policy and readiness to use the new, more effective artemisinin-based combination therapies (ACT) are crucial [17]. Along the north-western border of Thailand, where highly multi-drug resistant isolates of *P. falciparum *are found, artesunate-mefloquine combination therapy (MAS3) is the standard treatment regimen for uncomplicated falciparum malaria [9]. Early diagnosis and treatment with an artemisinin-based drug combination of very high efficacy that reduces gametocyte carriage, has led to a marked decline in the incidence of falciparum malaria and a reversal of the previous trend toward increasing mefloquine resistance [18]. Artemisinin derivatives will ensure rapid clinical and parasitological responses and are remarkably effective, hence clinical deterioration is extremely unusual. To optimize therapy, combination with a slower-acting antimalarial drug is required. Systematic use of ACT would help to delay the emergence of resistance if the drug was used widely. However, the continued use of mefloquine monotherapy or with only 2 days of artesunate in this region, provides persistent selective pressure to continue the evolution of mefloquine resistance, which could diminish the efficacy of the artesunate-mefloquine combination and that of artemether-lumefantrine.

Artemether-lumefantrine has been introduced recently for oral treatment of uncomplicated *falciparum *malaria. In Thailand, several trials have been conducted with this combination. The six-dose schedule provides sustained blood lumefantrine levels and thus improved cure rates [11]. Lumefantrine is highly lipophilic and the oral bioavailability varies considerably between individuals and increases greatly if the drug is administered after a meal rich in fat [19].

The present trial reconfirmed the efficacy of the six-dose regimen of artemether-lumefantrine given over three days [20]. Both treatments in this study cleared fever and parasitaemia promptly and reliably. Both treatments were well tolerated and highly effective. Importantly 2/3 less *P. vivax *infections and 12 days longer median time to appearance of *P. vivax *parasitaemia in the MAS3 group were most probably due to the longer terminal half-life of mefloquine compared to lumefantrine [21,22]. More data on the safety and efficacy of artemether-lumefantrine in very small children and pregnant women are needed.

## Authors' contributions

RH carried out the study and analyzed the data. RH, EAA, RMG, PS, TJ, NJW, FN conceived the study, participated in its design and co-ordination and contributed to draft the manuscript. LP, KLT assisted in collection of data. AB performed the PCR experiments. All authors read and approved the final manuscript.

**Table 2:** Treatment response.

Treatment group	ALN* (n = 245)	MAS3** (n = 245)
Compliance, no. (%)		
Completed day 7	241 (99.6%)	240 (99.2%)
Completed day 28	232 (95.9%)	233 (96.3%)
Completed day 42	225 (93.0%)	227 (93.4%)
Cumulative proportion of patients with clinical failure, no (%)		
Day 7	0 (0)	0 (0)
Day 28	13 (5.6)	14 (6.0)
Day 42	27 (12.0)	24 (10.6)
PCR, no.		
Novel	23	14
Recrudescent	2	8
Novel + recrudescent	1	1
Indeterminate/missing	1	1
PCR-adjusted cure rates, no. (%)		
Day 7	0 (100)	0 (100)
Day 28	2 (99.1)	9(96.1)
Day 42	3 (98.8)	9 (96.3)

* ALN = artemether-lumefantrine, ** MAS3 = artesunate-mefloquine
